# Systemic Inflammation Mediates the Association Between Admission Hyperglycemia and Pulmonary Infection or Prognosis in Acute Ischemic Stroke

**DOI:** 10.1155/mi/9595535

**Published:** 2026-03-18

**Authors:** Xiaojuan Ma, Jinwei Duan, Ye Cheng, Wu Li, Chen Liang, Tian Yang, Jie Liu, Yan Mi, Shan Du, Feixiao Xue, Gejuan Zhang, Mingze Chang, Wenzhen Shi, Ye Tian

**Affiliations:** ^1^ Clinical Medical Research Center, The Affiliated Hospital of Northwest University, Xi’an No. 3 Hospital, Xi’an, Shaanxi, China, xa3yuan.com; ^2^ Xi’an Key Laboratory of Cardiovascular and Cerebrovascular Diseases, The Affiliated Hospital of Northwest University, Xi’an No. 3 Hospital, Xi’an, Shaanxi, China, xa3yuan.com; ^3^ Department of Neurology, The Affiliated Hospital of Northwest University, Xi’an No. 3 Hospital, Xi’an, Shaanxi, China, xa3yuan.com; ^4^ Department of Clinical Laboratory, The Affiliated Hospital of Northwest University, Xi’an No. 3 Hospital, Xi’an, Shaanxi, China, xa3yuan.com

**Keywords:** acute ischemic stroke, mediation analysis, prognosis, stress-induced hyperglycemia, stroke-associated-pneumonia, systemic inflammation

## Abstract

**Background and Perspectives:**

Hyperglycemia frequently occurs after the onset of acute ischemic stroke (AIS) and the distinct effects and mechanisms of long‐term or transient hyperglycemia on stroke outcome are incompletely revealed. We aimed to investigate the potential mediating role of systemic inflammation in admission glycemia status with clinical outcome.

**Methods:**

A total of 2233 eligible AIS patients were enrolled from January 2018 to February 2024 and followed up for 12 months. Patients were stratified into three groups: normoglycemia (NG, *n* = 1341), persistent hyperglycemia (PHG, *n* = 588) and stress‐induced transient hyperglycemia (SIH, *n* = 304). Systemic immune‐inflammation index (SII), systemic inflammation response index (SIRI) were calculated from baseline blood cell counts. The primary outcomes were in‐hospital stroke‐associated pneumonia (SAP) and poor functional outcome (modified Rankin Scale [mRS] >2) at 12 months.

**Results:**

Among the included patients, 26.3% had PHG and 13.6% developed SIH. The rates of SAP and poor prognosis were highest in SIH group, intermediate in the PHG group and lowest in the NG group. Patients in SIH group exhibited highest systemic inflammatory levels (C‐reactive protein [CRP], hsCRP, lnSII, SIRI, and neutrophil‐to‐lymphocyte [NLR]). After adjusting for confounders identified by LASSO regression, both tertile 3 of lnSII and SIRI were significantly associated with higher risk of SAP independent of admission glycemia status. While higher lnSII and SIRI were significantly associated with poor prognosis at 12‐month only in SIH group. Mediation analysis demonstrated that lnSII partially mediated the association between glycemic status and clinical outcomes (mediation proportion: 16.7% for SAP; 10.8% for prognosis), with a similar effect observed for SIRI. The prediction model incorporating clinical variables and lnSII or SIRI yielded an AUC around 0.90 for SAP and 0.84 for 12‐month prognosis.

**Conclusion:**

Admission hyperglycemia, particularly SIH, notably affected the incidence of SAP and poor prognosis. Systemic inflammation partially mediated the effect of hyperglycemia on clinical outcome in AIS.

## 1. Introduction

Admission hyperglycemia is a common comorbidity occurring in ~40% of patients with acute ischemic stroke (AIS), which may arise from chronic dysglycemia (e.g., poorly controlled diabetes) or transient stress‐induced hyperglycemia (SIH), with the latter condition occurring in nondiabetic individuals or previously well‐controlled diabetics during acute illness [1]. The presence of admission hyperglycemia, particularly SIH, is associated with worse neurological and functional outcomes in AIS [2–5]. Therefore, many researchers advocated to place more emphasis on the prevention and control of SIH [6]. Whereas intensive medical therapies targeting to control admission hyperglycemia failed to counteract the risk of 90‐day poor prognosis of AIS in several random clinical trials [7, 8]. Thus, SIH may represent a heterogeneous pathological condition, and deep understanding on its role in affecting stroke outcomes is urgently needed in clinical practice to expand our option to address the SIH issue [9, 10].

Immune and inflammatory response play critical roles in the occurrence and progression of ischemic stroke [11]. Several cell experiments and animal studies revealed that hyperglycemia would trigger immune and inflammatory reactions in ischemic cerebral diseases [5, 12, 13]. Meanwhile, acute glucose fluctuation acts as a more critical factor in inducing neuron apoptosis and inflammation than continuous hyperglycemia in hippocampus of diabetic rats [14]. However, clinical studies on their associations in AIS are currently limited. Feng et al. [15] reported that neutrophil counts and neutrophil‐to‐lymphocyte (NLR) are positively associated with stress hyperglycemia ratio (SHR) in AIS patients. Considering the heterogeneous effects of persistent hyperglycemia (PHG) and transient SIH, their diverse effects on inflammation post stroke should be evaluated in more details.

Systemic immunity inflammation index (SII) and systemic inflammation response index (SIRI) are new composite biomarkers calculated from routine complete blood count (CBC) tests to effectively reflect body’s inflammatory status or balance between inflammation and immune status. They have gradually gained more attention for their cost‐effective and easily accessible characteristics as well as good predictive capabilities regarding various diseases [16]. Previous studies demonstrated that SIRI and SII exhibited better performance than the conventionally used NLR or platelet‐to‐lymphocyte (PLR) and comparable efficiency to C‐reactive protein (CRP) in predicting the clinical outcome of AIS [17, 18]. Few studies have examined the association between admission hyperglycemia and SII or SIRI, especially their effects on clinical outcome in AIS population. The present study will evaluate the contribution of admission glycemia status (categorized into normoglycemia [NG], PHG and SIH) to the incidence of in‐hospital stroke‐associated pneumonia (SAP) and unfavorable 12‐month outcome after adjusting main confounding factors. Further, we investigate the association between glycemia and inflammatory level by introducing SII and SIRI indices, and then quantified the mediating role of inflammation in the influence of admission hyperglycemia on clinical outcome.

## 2. Methods

### 2.1. Participates

The present study evaluated patients admitted for AIS based on data from a prospective cohort (ChiCTR‐EOC‐17012190) which recruited patients between Jan 2018 and Feb 2024. AIS was defined by a sudden neurological dysfunction caused by focal brain ischemia with imaging evidence of acute infarction [19]. The inclusion criteria of the study were: (1) aged over 18 years; (2) admitted to hospital within 3 days after the onset of stroke; (3) MRI or CT imaging confirmed AIS. The exclusion criteria were: (1) patients didn’t underwent glucose and HbA1c tests within 24 h after admission; (2) patients didn’t have blood cell counts test within 24 h after admission; (3) patients missed the 12‐month follow‐up; (4) severe hepatic or renal insufficiency; (5) patients with hematologic malignancies or severe solid tumors; (6) patients suffering acute trauma or underwent surgeries within a month before the onset of stroke; (7) patients with immune or infectious diseases or taking immunosuppressive medicines within 2 weeks prior to admission. This study was approved by the ethic committee of our hospital and performed in accordance with the declaration of Helsinki. Informed consents were obtained from patients or their caregivers.

### 2.2. Definition of Hyperglycemia

The eligible AIS patients were categorized into three groups, including NG, PHG and stress‐induced hyperglycemia (SIH) based on the admission glucose, HbA1c levels and history of diabetes. In current study, admission NG was defined as (a) HbA1*c* ≤ 6.5% and admission blood glucose (ABG) < 7.8 mmol/L in nondiabetic patients or (b) HbA1*c* ≤ 7.0% and ABG < 7.8 mmol/L in diabetic patients. PHG was defined as ABG ≥ 7.8 mmol/L with HbA1*c* > 6.5% in nondiabetic patients or HbA1*c* > 7.0% in diabetic patients. SIH was defined as a HbA1*c* ≤ 6.5% and ABG ≥ 7.8 mmol/L in nondiabetic patients or HbA1*c* ≤ 7.0% and ABG ≥ 7.8 mmol/L in diabetic patients. The definition of admission hyperglycemia with blood glucose concentration of >7.8 mmol/L is based on the standard by the American Diabetes Association and the American Association of Clinical Endocrinologists consensus [20]. The level of HbA1c serves as a reliable marker of the mean glucose concentration or glycemic control over the previous 3–4 months to distinguish the persistent and transient SIH with different cutoff between diabetic and nondiabetic patients [21, 22]. Diabetes diagnosed before admission was identified by either record of confirmed diagnosis of diabetes or prescription of oral or injectable antidiabetic medication. Moreover, the SHR was used to determine the presence and degree of stress hyperglycemia following a stroke accident and it was calculated as random blood glucose at admission (mmol/L)/estimated average glucose level (mmol/L) before admission, where the average glucose level was estimated by HbA1c using the formula of (1.59 × HbA1c) − 2.59 [23].

### 2.3. Systemic Inflammation Assessment

Lymphocyte, neutrophil, monocyte, and platelet counts were evaluated using automated hematology analyzing devices. Systemic immune‐inflammation index (SII) was calculated as platelet count × neutrophil count/lymphocyte count, and then was converted into natural logarithm form (lnSII). SIRI was measured using the formula of monocyte count × neutrophil count/lymphocyte count. CRP and hs‐CRP were evaluated using immunoturbidimetry method and they were abnormal when the values elevated above 10 and 3 mg/L, respectively.

### 2.4. Outcome Assessment and Follow‐Up

The primary clinical outcomes were the occurrence of SAP within the first 7 days after the onset of stroke and poor functional outcome at 12‐month of follow‐up. SAP was diagnosed based on standardized criteria developed by the US Centers for Disease Control and Prevention as described [24, 25]. Patients were followed up for functional outcomes via clinic interview in‐person or telephone communication at 12 months (±15 days). Functional outcome was assessed using the modified Rankin Scale (mRS) score, a measure of disability widely used for post‐stroke recovery with a score of 3–6 defined poor outcomes (functional dependance). All events were collected by trained research coordinators who were blinded to subjects’ baseline characteristics.

### 2.5. Statistical Analysis

The normally distributed variables were expressed as means ± standard deviation (SD) and the difference among three groups were compared using analysis of variance (ANOVA) followed by pairwise comparison using Bonferroni post‐hoc test. The skewed distributed variables were expressed as median with quartiles (Q25, Q75) and compared using nonparametric Kruskal–Wallis test following by post‐hoc all‐pairwise comparison with Bonferroni correction. Categorical variables were shown as frequencies (percentage) and compared using Chi‐square test. Pearson correlation test was performed to explore the correlation between glycemic parameters and inflammation level. LASSO regression with 10‐fold cross‐validation was carried out to filter important clinical variables associating with SAP or 12‐month poor prognosis using “glmnet” package to standardize and centralize the included variables, and obtain the best‐fit *λ* values at *λ*_1SE_. The input variables in the LASSO formula included demographic data, concurrent diseases conditions, laboratory parameters, admission mRS, National Institute of Health Stroke Scale (NIHSS), TOAST etiological subtypes, previous medications, and in‐hospital treatment strategies. The resultant variables were further adjusted as covariates in the adjusted logistic regression models, restricted cubic spline (RSC), ROC analysis and mediation analysis evaluating the association between glucose or inflammation and clinical outcomes. False discovery rate (FDR)‐Benjamini–Hochberg correction was performed for multiple testing in logistic analysis involving multiple outcomes (SAP, 12‐month prognosis) and systemic inflammation indices (lnSII, SIRI) or glycemic status. RCS analysis was performed to detect the nonlinear relationships between the SHR and the prevalence of SAP or poor prognosis after adjustment for covariates. The prerequisites for mediation analysis were defined as a significant indirect effect, a significant total effect, and a positive proportion of the mediator effect as previous described [26]. A “bruceR” package was utilized to assess the mediating effects of inflammatory indicators (lnSII and SIRI) on the associations between admission glycemia status and 12‐month prognosis, adjusted by the covariates. ROC analysis was used to evaluate the predicting efficiency of SHR, lnSII, SIRI as well as composite models adding clinical variables on SAP or prognosis. Then the potential benefits of these predictors and models were assessed using decision curve analysis (DCA) by comparing the net benefit against the strategies of treating all or no patients across a spectrum of threshold probabilities relevant to SAP or prognosis. We performed bootstrap resampling (*B* = 1000 iterations) on the training set to evaluate the internal stability of the model coefficients and performance metrics. For each iteration, a bootstrap sample was drawn with replacement, and the model was retrained and the AUC of each model was reported. All data were analyzed using SPSS26.0 software and R 4.2.1 version. Two‐tailed values of *p*  < 0.05 were considered statistically significant.

## 3. Results

### 3.1. Baseline Characteristics of the AIS Patients With Different Admission Glycemia Status

**Figure 1:**
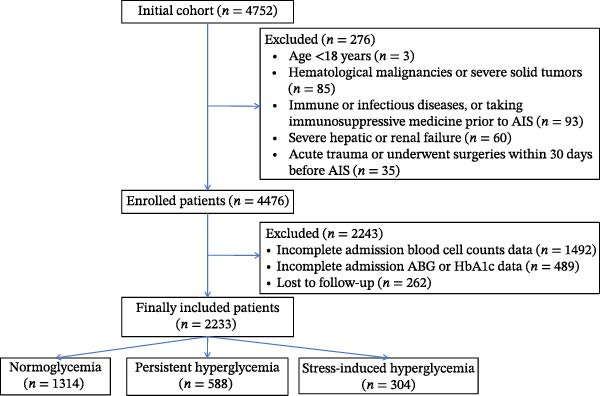
Flow chart of patient selection.

From an initial cohort of 4752 AIS patients, 276 patients were excluded for age and concurrent disease conditions, 2243 patients were excluded for incomplete blood cell counting or blood glucose data at admission, and 262 patients were lost in 1 year follow‐up. Finally, an overall of 2233 patients were included in this study (Figure 1). The baseline characteristics of the included and excluded patients were compared. There were no differences in admission glucose levels between the two groups, except for some secondary variables (Supporting Information 1: Table S1). The median age of the included patients was 65 (56, 73) years and 68.2% of them were male gender. Based on the admission glycemia status and diabetes history, the eligible patients were stratified into NG (*n* = 1341), PHG (*n* = 588) and SIH (*n* = 304) groups, with the PHG and SIH accounting for 26.3% and 13.6% of the whole population. There was statistically significant difference across the three groups in patient age, gender, medical history except stroke, admission stroke severity and multiple items of laboratory parameters involving lipid metabolism, glucose, coagulation, and blood cell counts (*p* < 0.05). The SIH group had higher age, baseline mRS and NIHSS, percentage of cardioembolic origin in TOAST classification and elevated atrial fibrillation or coronary heart disease than the other two groups. The PHG group had median HbA1c and glucose reaching 8.3% and 10.27 mmol/L, substantially higher than other groups (*p* < 0.05). SIH group had a peak value of SHR at 1.36 (1.19, 1.52) among the three groups. Diabetic history was recorded in 386 (65.6%) patients in PHG group and 81 (26.6%) patients in SIH group, respectively. Moreover, the PHG group had higher prevalence of dyslipidaemia with highest median TC, TG, and LDL. We also noticed that higher counts of white blood cell and neutrophils presented in SIH patients. In SIH group, there was a higher frequency of dysphagia, and more patients received thrombolysis or endovascular treatments than the other two groups (Table 1).

**Table 1:** Baseline characteristics of the AIS patients with different admission glycemia status.

Characteristics	Overall	Normoglycemia (*n* = 1341)	Persistent hyperglycemia (*n* = 588)	Stress‐induced hyperglycemia (*n* = 304)	*p*
Age (years)	65 (56, 73)	65 (56, 72)	65.5 (49.1, 65.5)	68 (56, 78)	<0.001
Gender (male, %)	1523 (68.2%)	945 (70.5%)	389 (66.2%)	189 (62.2%)	0.009
BMI (kg/m^2^)	24.22 (22.23, 26.12)	24.39 (22.49, 26.17)	24.09 (22.05, 26.06)	23.66 (21.77, 25.88)	0.855
Smoking	910 (40.8%)	561(41.8%)	243(41.3%)	106 (34.9%)	0.078
Alcohol	553 (24.8%)	343 (25.6%)	146 (24.8%)	64 (21.1%)	0.256
Medical history					
Hypertension	1394 (62.4%)	784 (58.5%)	408 (69.4%)	202 (66.4%)	<0.001
Diabetes	593 (26.6%)	126 (9.4%)	386 (65.6%)	81 (26.6%)	<0.001
Dyslipidaemia	451 (20.2%)	227 (16.9%)	183 (31.3%)	41 (13.5%)	<0.001
Atrial fibrillation	262 (11.8%)	144 (10.7%)	59 (10.0%)	61 (20.1%)	<0.001
Coronary heart disease	380 (17.0%)	197 (14.7%)	112 (19.0%)	71 (23.4%)	<0.001
Stroke	433 (19.4%)	244 (18.2%)	122 (20.7%)	67 (22.0%)	0.194
Admission mRS	2 (1, 4)	2 (1, 4)	2 (1, 4)	4 (2, 5)	<0.001
Admission NIHSS	3 (1, 8)	3 (1, 7)	3 (1, 7)	9 (3, 15)	<0.001
TOAST					<0.001
Large‐artery atherosclerosis	912 (46.4%)	518 (54.1%)	286 (54.1%)	108 (41.5%)	
Cardioembolism	287 (14.6%)	155 (13.2%)	56 (10.6%)	76 (29.2%)	
Small artery occlusion	673 (34.2%)	445 (37.8%)	167 (31.6%)	61 (23.5%)	
Other determined	27 (1.7%)	19 (1.6%)	4 (0.8%)	4 (1.5%)	
Undetermined	67 (3.4%)	40 (3.4%)	16 (3.0%)	11 (4.2%)	
Heart rate	75 (67, 84)	75 (66, 83)	76 (68, 86)	75 (70, 83)	<0.001
SBP (mmHg)	150 (135, 166)	148 (134, 164)	155 (140, 169)	152 (137, 168)	0.002
DBP (mmHg)	85 (76, 95)	84 (75, 95)	86 (77, 94)	85 (77, 93)	0.311
Laboratory parameters					
TC (mmol/L)	4.23 (3.49, 5.00)	4.20 (3.49, 4.95)	4.42 (3.56,5.33)	4.09 (3.18, 4.74)	<0.001
TG (mmol/L)	1.33 (0.90, 1.93)	1.26 (0.86, 1.79)	1.56 (1.10,2.53)	1.15 (0.78,1.69)	<0.001
LDL (mmol/L)	2.38 (1.88, 2.95)	2.35 (1.88, 2.93)	2.49 (1.95, 3.15)	2.31 (1.62, 2.78)	<0.001
HDL (mmol/L)	1.13 (0.95, 1.31)	1.14 (0.96, 1.32)	1.11 (0.94, 1.30)	1.10 (0.95, 1.31)	0.034
HbA1c (%)	5.9 (5.5, 6.9)	5.6 (5.3, 6.0)	8.3 (7.4, 9.63)	5.6 (5.3, 5.9)	<0.001
Glucose (mmol/L)	6.49 (5.46, 8.56)	5.7 (5.17, 6.44)	10.27(7.79, 12.60)	8.83 (8.34, 9.94)	<0.001
SHR	0.93 (0.82, 1.09)	0.90 (0.82, 1.00)	0.91 (0.77, 1.13)	1.36 (1.19, 1.52)	<0.001
Serum creatinine (U mol/L)	64 (54, 75)	64 (54, 75)	62 (54, 77)	63 (53, 79)	0.264
Homocysteine (mmol/L)	16 (12, 23)	16 (12, 24)	14 (12, 19)	16 (11, 24)	<0.001
Fibrinogen (mg/dL)	298 (260, 346)	293 (256, 342)	314 (271, 356)	304 (252, 346)	<0.001
FDP (μg/mL)	1.02 (0.55, 1.94)	0.94 (0.51, 2.01)	0.97 (0.61, 1.71)	1.42 (0.75, 2.93)	<0.001
D‐dimer (ng/mL)	384 (246, 834)	369 (230, 854)	368 (248, 738)	623 (295, 1145)	<0.001
WBC (×10^9^/L)	7.08 (5.83, 8.71)	6.8 (5.66, 8.41)	7.41 (6.10, 8.95)	7.53 (6.07, 9.62)	<0.001
RBC (×10^12^/L)	4.60 (4.15, 4.97)	4.55 (4.12, 4.92)	4.68 (4.26, 5.02)	4.60 (4.14, 4.91)	<0.001
Hemoglobin (g/L)	142 (129, 153)	142 (129, 153)	142 (129, 153)	141 (128, 152)	0.004
Neutrophils (×10^9^/L)	4.60 (3.60, 6.26)	4.40 (3.40, 5.91)	4.85 (3.73, 6.50)	5.34 (4.09, 7.24)	<0.001
Lymphocytes (×10^9^/L)	1.60 (1.17, 2.10)	1.60 (1.20, 2.10)	1.71 (1.20, 2.29)	1.39 (0.91, 1.96)	<0.001
Monocytes (×10^9^/L)	0.44 (0.34, 0.58)	0.44 (0.34, 0.57)	0.46 (0.37, 0.59)	0.45 (0.30, 0.61)	0.556
PLT (×10^9^/L)	195 (157, 238)	196 (156, 237)	197 (160, 242)	194 (152, 230)	0.125
Medical history					
Anti‐platelet	282 (12.7%)	162 (12.2%)	78 (13.4%)	42 (13.8%)	0.652
Anti‐coagulate	161 (7.2%)	88 (6.6%)	47 (8.0%)	26 (8.6%)	0.333
Anti‐hypertension	963 (43.3%)	526 (39.3%)	290 (49.7%)	147 (48.5%)	<0.001
Lipid lowering	210 (9.4%)	108 (8.1%)	60 (10.2%)	42 (13.8%)	0.006
Anti‐diabetes	444 (19.9%)	90 (6.7%)	299 (51.0%)	55 (18.4%)	<0.001
Dysphagia	258 (11.6%)	128 (9.5%)	57 (9.7%)	73 (24.0%)	<0.001
Awake stroke	201 (9.0%)	126 (9.4%)	46 (7.8%)	29 (9.5%)	0.507
Treatment					<0.001
Medical therapy	1352 (60.5%)	840 (62.6%)	380 (64.6%)	132 (43.4%)	
Iv‐tPA	481 (21.5%)	291 (21.7%)	117 (19.9%)	73 (24.0%)	
MT	287 (12.9%)	150 (11.2%)	63 (10.7%)	74 (24.3%)	
IV‐tPA+MT	113 (5.1%)	60 (4.5%)	28 (4.8%)	25 (8.2%)	

Abbreviations: BMI, body mass index; DBP, diastolic blood pressure; FDP, fibrinogen degradation products; HDL, high density lipoprotein; Iv‐tPA, intravenous thrombolysis; LDL, low density lipoprotein; mRS, modified Rankin scale; MT, mechanical thrombectomy; NIHSS, National Institutes of Health Stroke Scale; PLT, platelet; RBC, red blood cells; SBP, systolic blood pressure; SHR, stress hyperglycemia ratio; TC, total cholesterol; TG, triglyceride; WBC, white blood cells.

### 3.2. Multivariate Analysis of Admission Glycemia Status With Primary Clinical Outcomes

In the enrolled patients, 439 (19.7%) patients developed SAP within 7 days of disease onset and 671 (30.7%) patients had poor prognosis accounting at the 12‐month. The incidence of SAP and poor prognosis were highest in SIH, intermediate in PHG and lowest in NG group. In SIH group, 41.1% of the patients suffered from SAP and half of patients had poor prognosis at 12 months, which were significantly higher than PHG and NG groups (both *p*  < 0.05) (Table 2 and Supporting Information 2: Figure S1).

**Table 2:** Clinical outcomes of the stroke patients with different admission glycemia status.

Outcomes	Overall (*n* = 2233)	Normoglycemia (*n* = 1341)	Persistent hyperglycemia (*n* = 588)	Stress‐induced hyperglycemia (*n* = 304)	*p*
Stroke‐associated pneumonia	439 (19.7%)	204 (15.6%)	110 (18.7%)	125 (41.1%)	<0.001
In‐hospital mortality	57 (2.6%)	17 (1.3%)	9 (1.5%)	31 (10.2%)	<0.001
Hospital stay (days)	10 (8, 13)	10 (8, 13)	10 (8, 14)	11 (8, 15)	0.019
12‐month poor prognosis	671 (30.0%)	326 (24.3%)	193 (32.8%)	152 (50.0%)	<0.001
12‐month mortality	272 (12.2%)	118 (8.8%)	71 (12.1%)	83 (27.3%)	<0.001

The association of admission glycemia and SAP or prognosis was assessed using multivariate logistic regression. The potential covariates associating with clinical outcomes were screened using LASSO regression by inputting the baseline clinical characteristics as independent variables into the model. When the best‐fit *λ* values at *λ*_1SE_ were set, a total of 12 characteristics associating with SAP and 18 characteristics related to 12‐month poor prognosis were filtered (Supporting Information 3: Figure S2). To make the model more interpretable and briefer, the most influential variables on outcomes were selected based on the coefficient parameters and the focused variables in this study (SHR, diabetes, ABG, lnSII, SIRI, or CRP) were excluded from the confounder subsets. Therefore, the selected covariates associated with SAP were dysphagia, clinical treatments, admission mRS, NIHSS, TOAST etiological type, and age, while covariates related to prognosis were dysphagia, admission mRS, awake stroke, previous stroke history and mRS, NIHSS, age, and neutrophils count. These covariate subsets were further adjusted in the logistic regression analysis of the associations between admission glycemia status, SHR with pneumonia risk or prognosis, respectively. After adjustment for the confounders, SIH [1.764 (1.222–2.547), *p* = 0.005] significantly associated with the increased risk of SAP. PHG [1.532 (1.190–1.972), *p* = 0.004] and SIH [1.448 (1.071–2.068), *p* = 0.028] independently associated with the increased risk of poor prognosis. Both the second and third tertiles of SHR served as robust risk factors for SAP in the adjusted model, demonstrating a significant association of admission glycemia status with pneumonia risk in overall populations. The third tertile of SHR was independently associated with poor prognosis [1.390 (1.049–1.842), *p* = 0.029] (Table 3). Adjusted RSC regression analysis revealed that the risk of SAP and poor prognosis linearly increased with SHR (for nonlinearity, *p*  > 0.05) (Figure 2A,B).

Figure 2Restricted cubic spline (RCS) analysis of the relationship between SHR and the risk of SAP (A) or 12‐month poor prognosis (B) after adjustment for the covariates.

**Figure:**
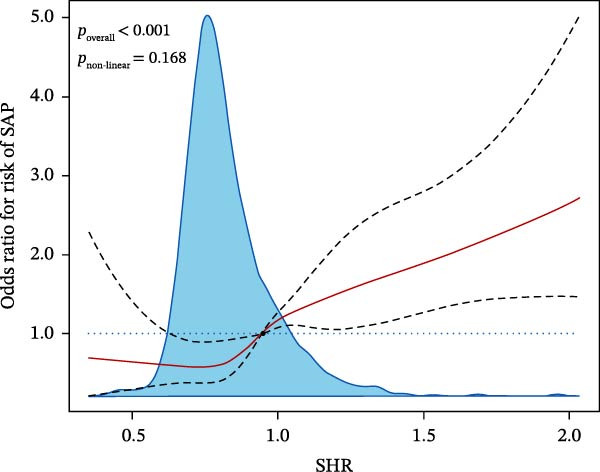
(A)

**Figure:**
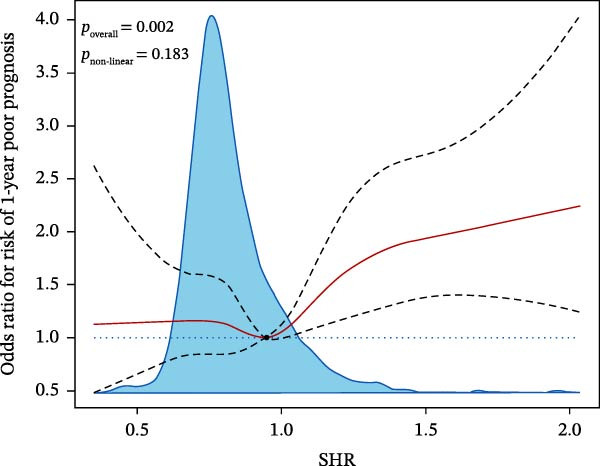
(B)

**Table 3:** Logistic analysis of the association between admission glycemia status with SAP or 12‐month prognosis.

Variable	SAP^a^			12‐month poor prognosis^b^		
	OR (95%CI)	*p*	*p* ^c^	OR (95%CI)	*p*	*p* ^c^
Crude model						
NG (*n* = 1034)	Reference	—	—	Reference	—	—
PHG (*n* = 588)	1.283 (0.994–1.656)	0.056	0.064	1.521 (1.230–1.882)	<0.001	0.001
SIH (*n* = 304)	3.892 (2.963–5.113)	<0.001	<0.001	3.113 (2.408–4.026)	<0.001	0.001
SHR	—	—	—	—	—	—
T1(≤0.86) (*n* = 745)	Reference	—	—	Reference	—	—
T2 (0.87–1.05) (*n* = 744)	2.485 (1.780–3.467)	<0.001	<0.001	1.242 (0.978–1.577)	0.076	0.076
T3 (≥1.06) (*n* = 744)	6.532 (4.784–8.918)	<0.001	<0.001	2.636 (2.102–3.306)	<0.001	0.001
Adjusted model						
NG (*n* = 1034)	Reference	—	—	Reference	—	—
PHG (*n* = 588)	1.389 (1.007–1.914)	0.045	0.051	1.532 (1.190–1.972)	0.001	0.004
SIH (*n* = 304)	1.764 (1.222–2.547)	0.002	0.005	1.448 (1.071–2.068)	0.018	0.028
SHR	—	—	—	—	—	—
T1 (≤0.86) (*n* = 745)	Reference	—	—	Reference	—	—
T2 (0.87–1.05) (*n* = 744)	1.642 (1.100–2.449)	0.015	0.029	0.974 (0.735–1.292)	0.856	0.856
T3 (≥1.06) (*n* = 744)	2.897 (1.993–4.212)	<0.001	0.002	1.390 (1.049–1.842)	0.022	0.029

Abbreviations: NG, normoglycemia; PHG, persistent hyperglycemia; SAP, stroke associated pneumonia; SHR, stress hyperglycemia ratio; SIH, stress induced hyperglycemia; T1/2/3, tertile 1, 2, 3.

^a^Adjusted for age, admission mRS and NIHSS, TOAST subtypes, clinical treatments, and dysphagia.

^b^Adjusted for age, previous stroke history and mRS, admission mRS and NIHSS, awake stroke, neutrophils count, and dysphagia.

^**c**^Benjamini–Hochberg FDR correction for multiple testings.

### 3.3. The Association Between Systemic Inflammatory Level and Admission Glycemia as Well as Primary Clinical Outcomes

**Figure 3:**
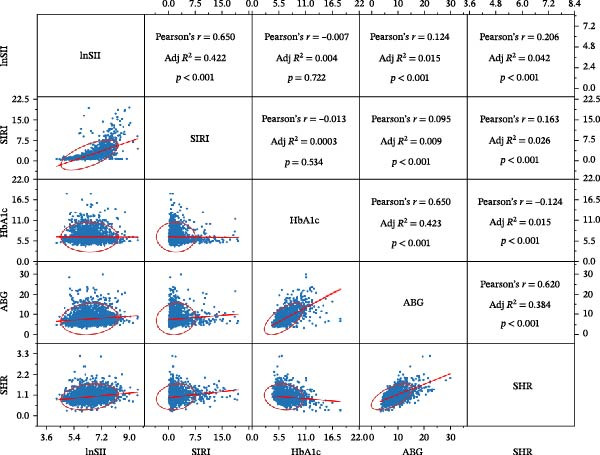
Correlation scatterplot matrix and corresponding coefficient between inflammatory indices (lnSII and SIRI) and glycemic parameters (SHR, HbA1c, and admission blood glucose) of the included patients.

To investigate the association between admission glycemia and systemic inflammatory level, *Pearson* correlation was used to analyze the relation among ABG, HbA1c, SHR, lnSII, and SIRI. The result revealed that ABG and SHR were closely associated with lnSII (*r* = 0.124, *p*  < 0.001; *r* = 0.206, *p*  < 0.001) or SIRI (*r* = 0.095 *p*  < 0.001; *r* = 0.163, *p*  < 0.001) (Figure 3). Both lnSII and SIRI stepwise increased in NG, PHG, and SIH groups with significant difference (*p* < 0.001). In PHG groups, 36.3% and 15.8% of the patients had abnormal hsCRP and CRP, with SIH group had the highest frequency (44.3% and 22.5%) than other groups (Table 4).

**Table 4:** Systemic inflammatory levels of the patients with different admission glycemia status.

Characteristics	Overall	Normoglycemia (*n* = 1341)	Persistent hyperglycemia (*n* = 588)	Stress‐induced hyperglycemia (*n* = 304)	*p*
hsCRP ≥ 3 mg/dL	613 (36.3%)	333 (33.8%)	164 (36.9%)	116 (44.3%)	0.007
CRP ≥ 10 mg/dL	266 (15.8%)	131 (13.6%)	74 (16.7%)	59 (22.5%)	0.002
SII	552.32 (350.81, 972.01)	519.27 (339.83, 882.22)	545.73 (315.31, 954.97)	768.4 (402.93, 1531.20)	<0.001
lnSII	6.31 (5.86, 7.46)	6.25 (5.83, 6.78)	6.30 (5.86, 6.86)	6.64 (5.99, 7.33)	<0.001
SIRI	1.29 (0.82, 2.21)	1.21 (0.78, 2.07)	1.33 (0.83, 2.21)	1.61 (1.03, 3.56)	<0.001
NLR	2.88 (1.91, 4.86)	2.71(1.85, 4.38)	2.87(1.95, 4.69)	3.99 (2.29, 8.06)	<0.001

Abbreviations: CRP, C‐reactive protein; Hs‐CRP, high‐sensitivity C‐reactive protein; NLR, neutrophil to lymphocyte ratio; SII, systemic immune‐inflammation index; SIRI, systemic inflammation response index.

**Figure 4:**
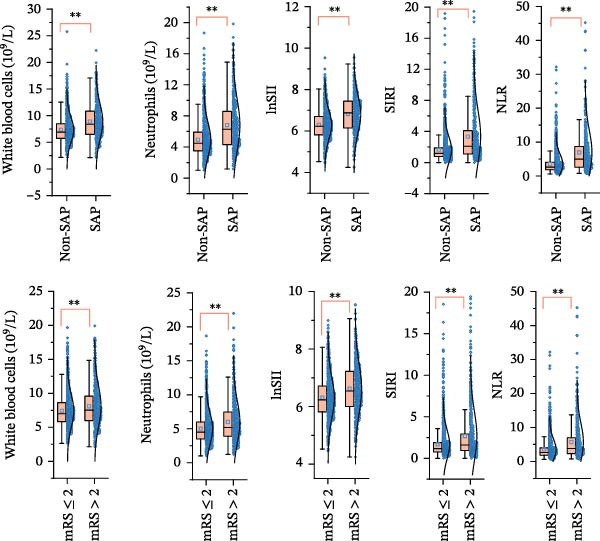
Box and scatter plot of inflammatory response indices between SAP and non‐SAP (upper panels) or good prognosis (mRS ≤ 2) and poor prognosis (mRS > 2).

We then investigated the association between inflammatory indices and clinical outcome, it was shown that patients suffered from SAP or poor prognosis had significantly higher admission WBC and neutrophil counts and lnSII, SIRI, and NLR than the corresponding control groups (Figure 4). In the adjusted model, both T3 of lnSII and SIRI served as robust factors associating with SAP [2.636 (1.851–3.753), *p* = 0.002; 2.934 (2.069–4.160), *p* = 0.002] and poor prognosis [1.409 (1.064–1.866), *p* = 0.034; 1.532 (1.161–2.021), *p* = 0.008] (Table 5). RSC analysis showed the nonlinear relationship between lnSII or SIRI and SAP (Figure 5). As stratified by admission hyperglycemia conditions, the T3 of lnSII or SIRI were significantly associated with SAP in the crude model and adjusted model almost in all the subsets, and patients with SIH had higher risk of SAP (Supporting Information 1: Table S2). While the higher inflammation markers only associated with 12‐month poor prognosis in patients with SIH (Supporting Information 1: Table S3).

Figure 5RCS analysis of the relationship between lnSII or SIRI and the risk of SAP (A,B) or 12‐month poor prognosis (C,D) after adjustment for the covariates.

**Figure:**
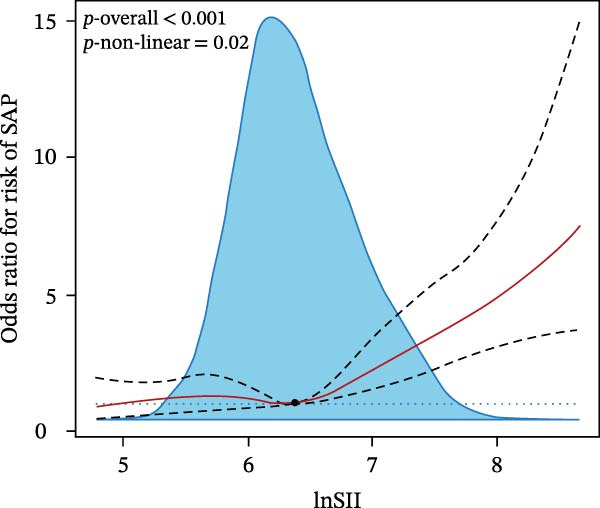
(A)

**Figure:**
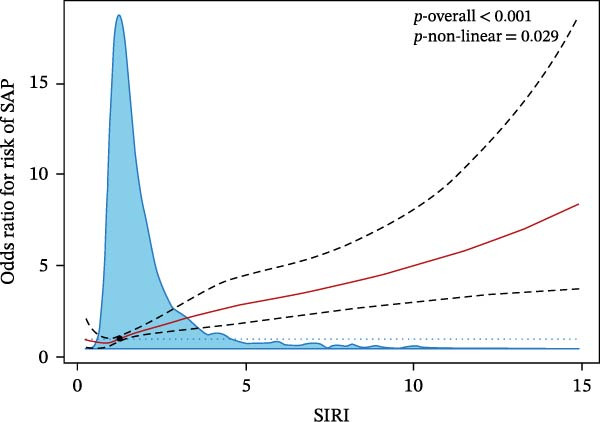
(B)

**Figure:**
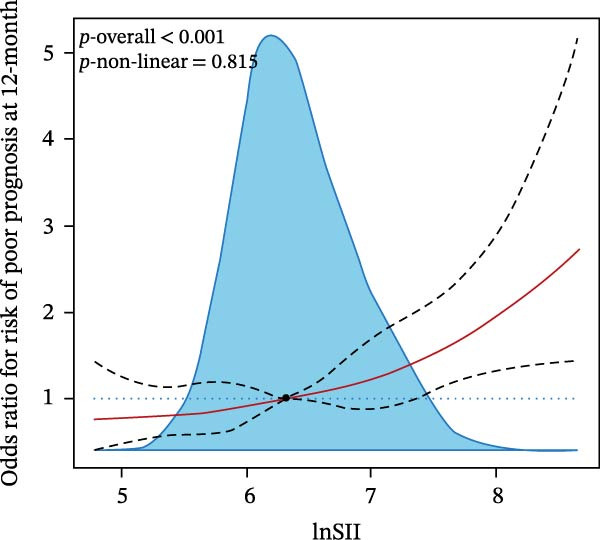
(C)

**Figure:**
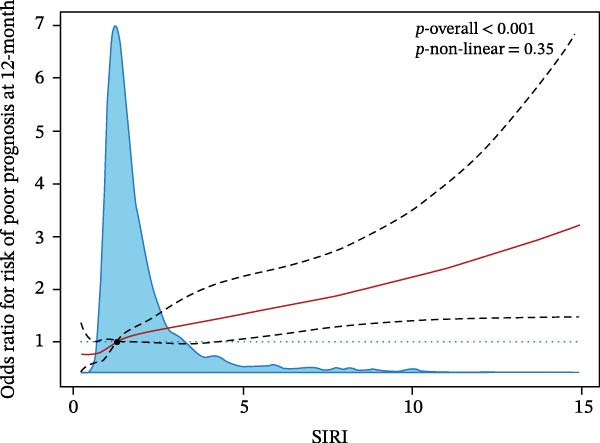
(D)

**Table 5:** Logistic analysis of the association between systemic inflammation with SAP or prognosis.

Variable	SAP^a^			12‐month poor prognosis^b^		
	OR (95% CI)	*p*	*p* ^c^	OR (95% CI)	*p*	*p* ^c^
Crude model						
lnSII	—	—	—	—	—	—
T1 (≤6.004) (*n* = 745)	Reference	—	—	Reference	—	—
T2 (6.005–6.653) (*n* = 744)	1.222 (0.896–1.665)	0.205	0.273	1.210 (0.955–1.532)	0.114	0.182
T3 (≥6.654) (*n* = 744)	4.103 (3.124–5.391)	<0.001	0.002	2.350 (1.876–2.943)	<0.001	0.002
SIRI	—	—	—	—	—	—
T1 (≤0.951) (*n* = 745)	Reference	—	—	Reference	—	—
T2 (0.952–1.781) (*n* = 744)	1.146 (0.843–1.558)	0.385	0.440	1.078 (0.850–1.368)	0.535	0.535
T3 (≥1.782) (*n* = 744)	3.824 (2.921–5.006)	<0.001	0.001	2.383 (1.906–2.980)	<0.001	0.002
Adjusted model						
lnSII	—	—	—	—	—	—
T1 (≤6.004) (*n* = 745)	Reference	—	—	Reference	—	—
T2 (6.005–6.653) (*n* = 744)	1.044 (0.704–1.547)	0.831	0.831	1.187 (0.897–1.570)	0.230	0.317
T3 (≥6.654) (*n* = 744)	2.636 (1.851–3.753)	<0.001	0.002	1.409 (1.064–1.866)	0.017	0.034
SIRI	—	—	—	—	—	—
T1 (≤0.951) (*n* = 745)	Reference	—	—	Reference	—	—
T2 (0.952–1.781) (*n* = 744)	1.265 (0.857–1.867)	0.238	0.317	1.101 (0.831–1.460)	0.501	0.572
T3 (≥1.782) (*n* = 744)	2.934 (2.069–4.160)	<0.001	0.002	1.532 (1.161–2.021)	0.003	0.008

Abbreviations: SAP, stroke‐associated pneumonia; SII, systemic immune‐inflammation index; SIRI, systemic inflammation response index.

^a^Adjusted for age, admission mRS and NIHSS, TOAST subtypes, clinical treatments, and dysphagia.

^b^Adjusted for age, previous stroke history and mRS, admission mRS and NIHSS, awake stroke, neutrophils count, and dysphagia.

^c^Benjamini–Hochberg FDR correction for multiple testings.

### 3.4. Mediation Analysis of the Inflammatory Indices in Affecting the Association Between Admission Glycemic Status on Clinical Outcomes

As illustrated in Figure 6A,B, significant and positive association between admission glycemia status (X) and lnSII or SIRI (M, path a) were revealed in effecting SAP (Y) after adjusting the covariates. The association between lnSII/SIRI and SAP (path b) was also positive and significant (*p* < 0.001). The path c′ indicated significant direct effects for the association between admission glycemia status and SAP (*p* < 0.01). The indirect effects (path a × b) of lnSII and SIRI were significant for SAP, explaining 16.7% and 12.0% of total variance (c), respectively. When evaluating the mediating effect of lnSII or SIRI in the effect of admission glycemia status on 12‐month poor prognosis, significant associations were also demonstrated between lnSII or SIRI with X and Y, as well as the direct and indirect effect pathways. The mediating effects of lnSII and SIRI explained 9.75% and 7.3% of total variance for poor prognosis (Figure 6C,D). Therefore, both direct and indirect effects showed systemic inflammatory response partially mediated the relationship between admission glycemia status and clinical outcomes.

Figure 6Mediation analysis of association with admission glycemia status with SAP mediated by lnSII and SIRI (A,B) and 12‐month poor prognosis (C,D). The models were adjusted for the covariates filter by LASSO regression.

**Figure:**
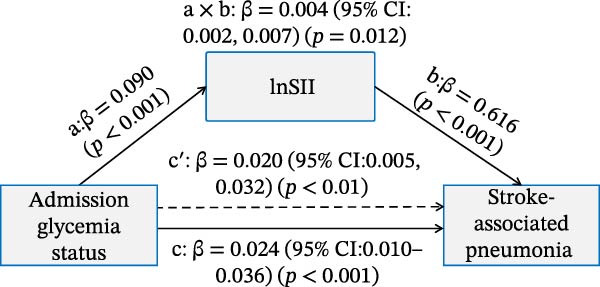
(A)

**Figure:**
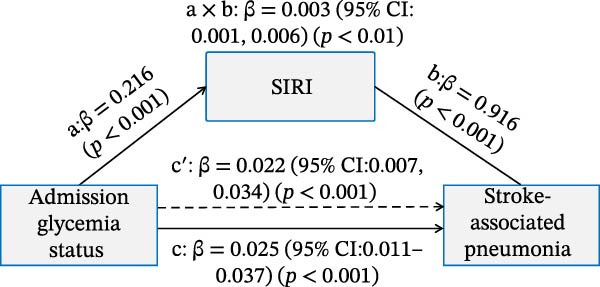
(B)

**Figure:**
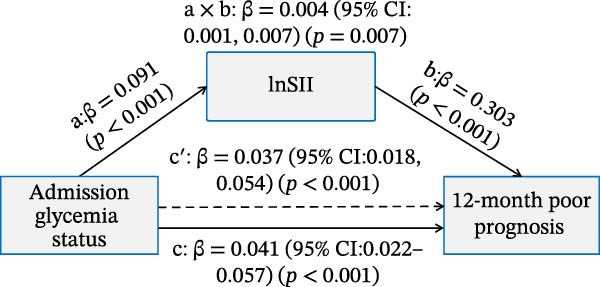
(C)

**Figure:**
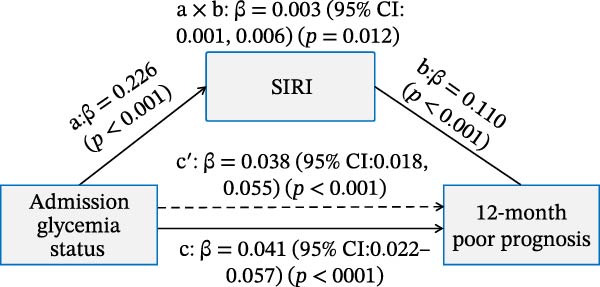
(D)

### 3.5. Predictive Values of SHR, lnSII, and SIRI for SAP and Long‐Term Prognosis of AIS

The performance of individual indices (SHR, lnSII, and SIRI) and their combination with important clinical variable subsets filtered by LASSO associating with SAP or 12‐month poor prognosis was evaluated using ROC analysis in the entire dataset. As depicted in Figure 7A, the admission SHR, lnSII, and SIRI indices predicted SAP with an AUC of 0.711, 0.682, and 0.680, respectively. However, when incorporating the clinical variables subset into the prediction models, their predictive performance greatly improved with an AUC around 0.90. The composite predictive model constructed using only clinical variables achieved an AUC of 0.895, indicating that individual SHR, lnSII, and SIRI indices didn’t show superiority in predicting the occurrence of SAP. The DCA curve showed that the lnSII and SIRI composite models predicting SAP demonstrated superior net benefit between 0.1 and 0.8 of the risk thresholds. Within this interval, these curves lie above both the “treat all” line and the “treat none” line, and this range is clinically meaningful because it covers a broad spectrum of decision thresholds commonly encountered in practice (Figure 7B). Bootstrap (*B* = 1000) based internal validation yielded congruent predicting performance with the initial training set for individual predictors or composite models, supporting the reproducibility and stability of the models (Figure 7C). With regarding to the 12‐month prognosis prediction, the three individual indices exhibited no discernible differences in predicting the risk of poor outcome with an AUC about 0.62, which increased to 0.84 when the related clinical baseline variables were added (Figure 7D). Within the threshold probability range between 0.1 and 0.9, the composite models showed higher net benefit than the “treat all” or “treat none” strategies (Figure 7E). The bootstrap resampling results confirmed the stability of composite models (all AUC around 0.82) comparing with those obtained from training set (Figure 7F).

Figure 7Predicting performance of SHR, lnSII, SIRI and clinical composite models of SAP and poor prognosis. ROC curve analysis (A) and DCA curves (B) evaluating SAP prediction of glycemic parameters alone, inflammatory indices alone, or their combination with clinical variables in the initial entire cohort. (C) Bootstrap (*B* = 1000) resampling validation of the prediction performance of the above parameter and composite models in the entire training set. (D–F) ROC, DCA curve and bootstrap evaluating of the performance of the models in 12‐month poor outcome prediction.

**Figure:**
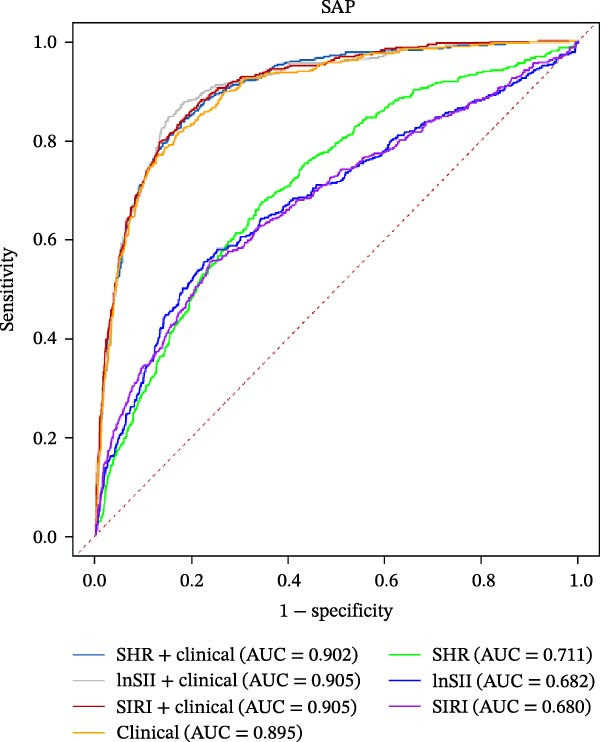
(A)

**Figure:**
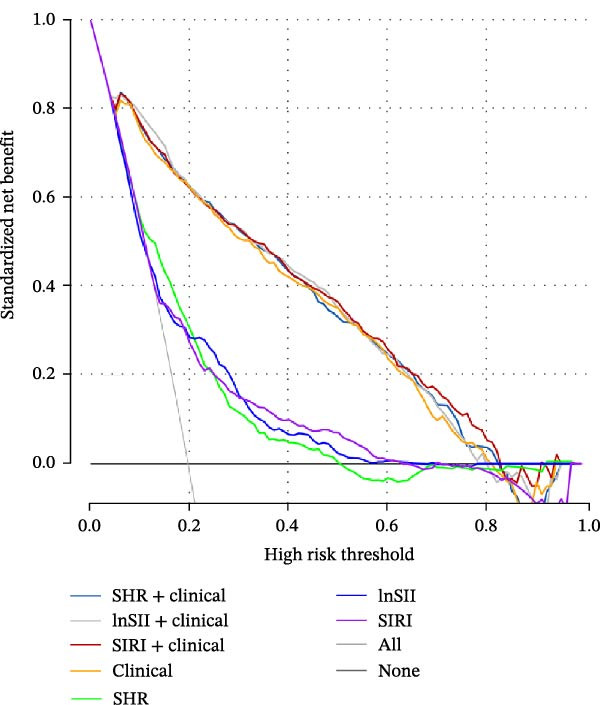
(B)

**Figure:**
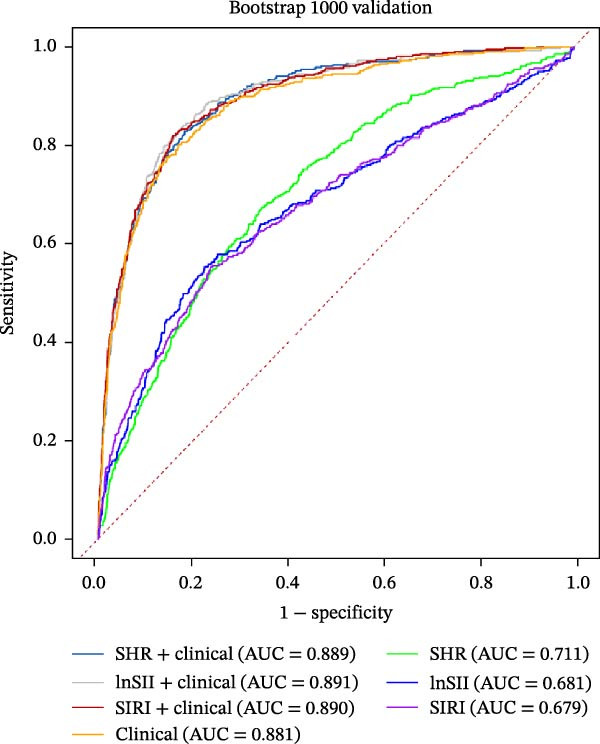
(C)

**Figure:**
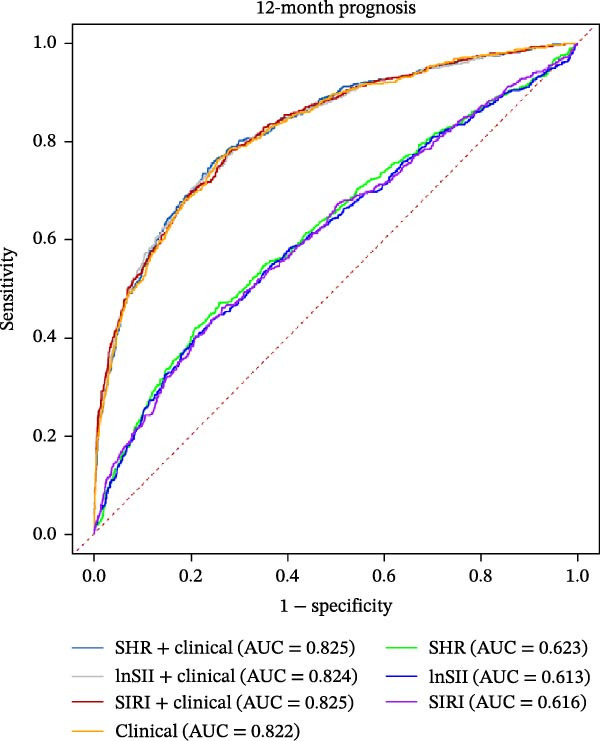
(D)

**Figure:**
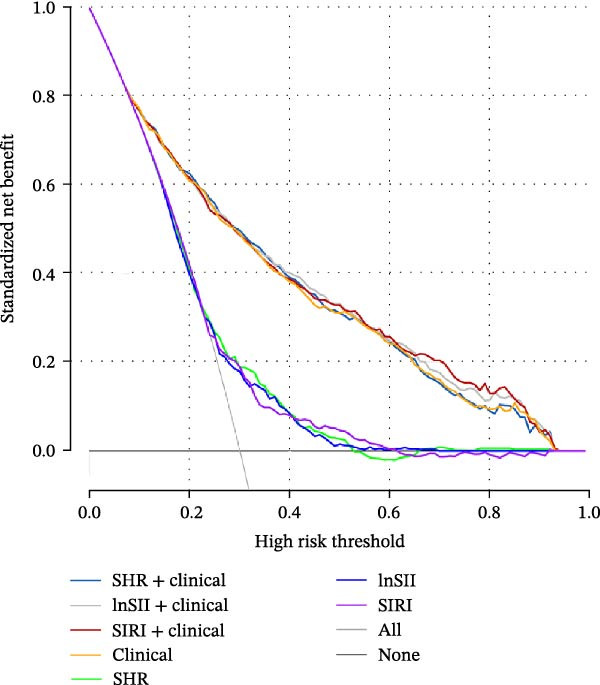
(E)

**Figure:**
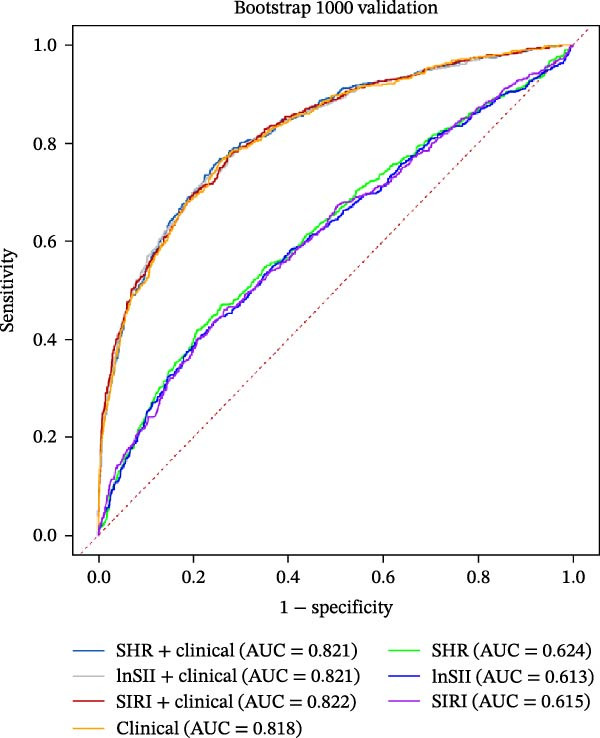
(F)

## 4. Discussion

Our study demonstrates that admission glycemia status, particular for SIH, is independently associated the susceptibility to SAP and poor functional outcomes in patients with AIS. Meanwhile, systemic inflammatory‐immune reaction mediates the effect of SIH in exacerbating cerebral injury and the related complications. This study first distinguishes hyperglycemia into PHG and transient SIH, and then linked the glycemia status with differential inflammatory profiles in AIS patients, which may provide novel mechanistic insights for SIH and a new perspective for addressing “glycemia control paradox” in clinical practice.

Hyperglycemia could manifest as chronic increase or poor control of blood glucose, or else acute glucose increase after disease onset. In our study, 39.5% of the included patients experienced admission hyperglycemia with PHG and SIH separately accounting for 25.9% and 13.6%. SIH group contained predominately nondiabetic patients (73.4%) and diabetic patients with previously well‐controlled glucose (26.6%), which was consistent with the opinion that although a diagnosis of SIH is usually reserved for patients without a history of diabetes, but patients with diabetes can still have a SIH response [22]. Currently, there is no uniform definition for SIH across different diseases, and a cut‐off value of admission blood glucose at 7.8 mmol/L (140 mg/dL) is mostly used in AIS patients as encapsulated by Yao et al. [27]. Then, the concepts of SHR, glycemia gap or glucose variability have been proposed to quantify the severity of SIH. However, these parameters may partially conceal the glycemia background information of the patients without considering pre‐existing diabetes and condition of glucose control. Therefore, our study distinguished hyperglycemia into persistent or transient status and incorporated the SHR index to depict the association between glycemia status and clinical outcomes, which took background glucose levels and diabetic history into account and facilitated for timely identification of patients with hyperglycemia requiring intervention in clinical practice [28].

The admission hyperglycemia had remarkable impacts on the clinical outcome of patients with AIS. SAP is one of the severe complications post‐stroke and occurs in 7.1%–38% of the patients [24]. Patients with hyperglycemia exhibit a significantly higher prevalence of SAP compared to normoglycemic couterparts during AIS [29]. Zonneveld et al. [30] reported that admission hyperglycemia was associated with post‐stroke infection and worse functional outcome after adjustment for potential confounding variables in nondiabetic AIS patients, rather than in the recorded diabetes mellitus. In our study, the incidence of SAP was 18.7% in PHG and sharply increased to 41.1% in patients with SIH. The robust association between SHR and 90‐day or 6‐month prognosis has been demonstrated, while the long‐term effect of SIH on functional disability are incompletely revealed [31]. In this study, the patients were followed 12‐month to evaluate the relative long‐term effect of SIH on life quality. We found that patients with SIH had significantly higher ratio of functional dependence and mortality than those in PHG group. After adjusting the covariates, admission SIH was associated with higher risk of 12‐month poor prognosis. These results highlighted the important contribution of SIH to the risk of SAP and poor prognosis in patients with AIS and its contribution may vary as per the glycemia conditions. The adverse effects of admission SIH have been recognized and appeal more attention for glycemic control in clinical practice. Nevertheless, the current evidence doesn’t support using intensive glucose control in these subjects due to their insufficiency in reducing the risk of mortality or improving prognosis in AIS patients. It is proposed that SIH may reflect a multiplicity of other pathological conditions, such as adrenergic and renin–angiotensin system over‐activity, hyperglucagonaemia, and pancreatic β‐cell dysfunction, which are incompletely resolved by insulin therapy and so worsen the prognosis [9]. Our study linked the hyperglycemia with systemic inflammation and confirmed that patients experiencing SIH confronted greater challenges for inflammatory reactions than those with PHG in AIS. Although a few clinical studies have reported the association between admission hyperglycemia and inflammatory response, they don’t dig into the distinct effect between persistent and acute hyperglycemia [15, 32]. An animal study provided clues for this relation and showed that acute glucose fluctuation acted as a more critical factor in inducing neuron apoptosis and inflammation than continuous hyperglycemia in hippocampus of diabetic rats [14]. However, it still needs further investigation regarding the regulating mechanism between acute hyperglycemia and inflammation.

With regarding to the contribution of inflammation in linking hyperglycemia with clinical outcome, a mediation analysis was first used in our study to reveal the significant mediating role of inflammation in delivering the effect of admission hyperglycemia. Our data showed that higher lnSII and SIRI tertiles were closely related to the risk of SAP in all patients, which was consensus with the previous reports [33, 34]. After the onset of AIS, patients were exposed to a pro‐inflammatory yet immunosuppressed state and predisposed to pulmonary infections [35, 36]. Elevated blood glucose levels impair immune function, including neutrophil activity, degranulation and phagocytic efficiency, and NET formation, increasing susceptibility to infections [37]. Patients with admission hyperglycemia, particular SIH, had higher levels SII or SIRI and risk of SAP. We also noticed that SII and SIRI were significantly associated with long term poor prognosis only in SIH patients. The long‐term prognosis of PHG patients can be affected by other factors, such as microvascular complications, insulin resistance, diabetes related comorbidities, or metabolic syndrome, while the prognosis of SIH patients is more directly related to the acute inflammation. In addition, PHG patients exhibit long‐term tolerance and adaptive immunological mechanism to the metabolic imbalance and glucose fluctuation, in turn having lower risk of poor prognosis than SIH [28, 38]. Considering the important mediating role of inflammation, the sole upstream glucose control can’t fully address the issue of inflammation, which may explain why intense glycemic control doesn’t improve the prognosis of AIS. Based on this rational, several anti‐inflammatory medications are being tested in acute phase of AIS while very limited data are available from these trials so far. The CANTOS study (Canakinumab anti‐inflammatory thrombosis outcomes study) laid foundation for investigating the effect of systemic inflammation suppression using IL‐1 neutralizing antibody with Canakinumab and showed clear benefit for patients with established atherosclerotic disease and high‐risk cardiovascular profile [39]. Current evidence supports the beneficial effect of TLR4 antagonist in patients underwent endovascular treatment [40] and the secondary prevention effect of long‐term colchicine for recurrent vascular events in noncardioembolic stroke [41, 42]. It is regretful that the RCT study NCT04123067, launched in 2020 to evaluate the effect of Pioglitazone on peripheral immune‐stress response in hyperglycemic stroke patients, has not updated the clinical data yet for unknown reason. Therefore, further clinical studies are needed to test the safety and efficiency of anti‐inflammatory approaches, particular for patients with admission hyperglycemia. It is also useful to make risk stratification according to systemic inflammatory level and glycemia status to identify who are prone to poor prognosis and benefit from anti‐inflammatory intervention. The present study has several limitations. First, the research was conducted in a single center, and the individual difference and disease severity variation would inevitably produce selection bias and limit the generalization of our findings. Second, we did not assess long‐term glycemic variability or specific inflammatory cytokines during hospitalization, which hindered our constantly assessment and deep understanding on SIH when the stress was relieved. Third, the design of this study was observational, and a cause–effect relationship between hyperglycemia and inflammation cannot be determined. Therefore, further randomized large‐scale studies are expected to validate our findings. Meanwhile, the causality of hyperglycemia and inflammation exposing chronic and acute glycemia insults should be explored in more details in animal model.

## 5. Conclusion

Our study demonstrated that admission hyperglycemia appeared in ~40% of the patients with AIS, and it independently associated with SAP and 12‐month poor prognosis. Patients suffering SIH were challenged with higher systemic inflammation levels reflected by the SIRI and SII indices. The tertile 3 of SIRI and SII were closely related to SAP in the overall population regardless of the glycemia status. Inflammation reaction partially mediated the effects of admission hyperglycemia on SAP or prognosis. Integrating these biomarkers into early risk stratification may help tailor preventive strategies, ultimately reducing infection‐related morbidity and improve outcome in this vulnerable population.

## Author Contributions

**Ye Tian, Wenzhen Shi, Mingze Chang and Gejuan Zhang:** conceptualization, project administration and supervision and manuscript revision. **Xiaojuan Ma:** writing – original draft. **Ye Cheng, Wu Li, Chen Liang and Tian Yang:** experiment and data collection. **Jie Liu, Yan Mi, Shan Du and Feixiao Xue:** literature review, investigation and visualization. **Xiaojuan Ma and Jinwei Duan:** statistical analysis of the data.

## Funding

This work was supported by grants from National Natural Science Foundation of China (Grant 82104155), Key Research and Development Program of Shaanxi (Grant 2024SF‐YBXM‐034), Xi’an Science and Technology Planning Project (Grants 24YXYJ0035 and 24YXYJ0100), Medical Research Programme of Xi’an No. 3 Hospital (Grants Y2023yxyj02 and Y2023qn0002), the Youth Talent Support Program of Xi’an Science and Technology Society (Grant 959202413020), Xi’an Yingcai Program 2024 (Grant XAYC240061), and Scientific Research Project of Xi’an Municipal Health Commission (Grant 2025ms17).

## Disclosure

All authors have read and approved the final manuscript.

## Ethics Statement

The studies involving human participants were reviewed and approved by the Ethics Committee of Xi’an No. 3 Hospital and in line with the principles of the Declaration of Helsinki. The patients or their caregivers provided their written informed consents.

## Consent

The authors have nothing to report.

## Conflicts of Interest

The authors declare no conflicts of interest.

## Supporting Information

Additional supporting information can be found online in the Supporting Information section.

## Supporting information


**Supporting Information 1** The supporting information provide further data analysis on the included patients and subgroup logistic analysis between systemic inflammation and clinical outcome to support the main analysis. Table S1: Comparison of baseline characteristics between the included and excluded patients. Table S2: Logistic analysis of the association between systemic inflammation level with the risk of SAP in various glycemia conditions. Table S3: Logistic analysis of the association between systemic inflammation level with the risk of 12‐month poor prognosis in various glycemia conditions.

**Supporting Information 2** Figure S1: mRS distribution at 12‐month follow‐up in acute ischemic stroke patients stratified by admission glycemic status.

**Supporting Information 3** Figure S2: Clinical covariates selection for SAP or poor prognosis based on LASSO regression model. (A) The coefficient paths of all baseline clinical variables for SAP across a sequence of increasing log (λ) values, generated via 10‐fold cross‐validation. Each colored line represents the trajectory of a standardized coefficient for one variable. (B) Cross‐validation for optimal λ selection in LASSO regression for SAP. The mean cross‐validated deviance is plotted against log (λ). The left and right dashed vertical line marks the λ value at λ_1min_ and λ_1s_, and the number of nonzero coefficients at each λ is shown along the top axis. (C) Predictor importance in the final sparse LASSO model. Horizontal bar plot displaying the standardized coefficients of the 8 features retained by the LASSO regression at the optimal λ1se. Variables are ranked by the absolute coefficients with red bar indicating a positive association with SAP while blue bar indicating an inverse association. (D–F) The procedure of feature selection associating with 12‐month poor prognosis using the same approach as above.

## Data Availability

The data that support the findings of this study are available from the corresponding author upon reasonable request.

## References

[1] Stalikas N. , Papazoglou A. S. , and Karagiannidis E. , et al.Association of Stress Induced Hyperglycemia With Angiographic Findings and Clinical Outcomes in Patients With ST-Elevation Myocardial Infarction, Cardiovascular Diabetology. (2022) 21, no. 1, 10.1186/s12933-022-01578-6, 140. 35883091 PMC9327277

[2] Klapproth S. , Meyer L. , and Kniep H. , et al.Delayed Neurological Recovery in Ischemic Stroke Patients Undergoing Endovascular Treatment Is Associated With Baseline Hyperglycemia: A Treatable Cause of the Stunned Brain Phenomenon?, Journal of Neurology. (2025) 272, no. 4, 10.1007/s00415-025-13019-x, 313. 40183971 PMC11971131

[3] Tanaka K. , Yoshimoto T. , and Koge J. , et al.Detrimental Effect of Acute Hyperglycemia on the Outcomes of Large Ischemic Region Stroke, Journal of the American Heart Association. (2024) 13, no. 23, 10.1161/JAHA.124.034556, e034556. 39575760 PMC11681584

[4] Rinkel L. A. , Nguyen T. T. M. , and Guglielmi V. , et al.High Admission Glucose Is Associated With Poor Outcome After Endovascular Treatment for Ischemic Stroke, Stroke. (2020) 51, no. 11, 3215–3223, 10.1161/STROKEAHA.120.029944. 33054674

[5] Peng Z. , Song J. , and Li L. , et al.Association Between Stress Hyperglycemia and Outcomes in Patients With Acute Ischemic Stroke due to Large Vessel Occlusion, CNS Neuroscience & Therapeutics. (2023) 29, no. 8, 2162–2170, 10.1111/cns.14163. 36914967 PMC10352867

[6] Zhang H. , Yue K. , and Jiang Z. , et al.Incidence of Stress-Induced Hyperglycemia in Acute Ischemic Stroke: A Systematic Review and Meta-Analysis, Brain Sciences. (2023) 13, no. 4, 10.3390/brainsci13040556, 556. 37190521 PMC10136900

[7] Johnston K. C. , Bruno A. , and Pauls Q. , et al.Intensive vs. Standard Treatment of Hyperglycemia and Functional Outcome in Patients With Acute Ischemic Stroke: The SHINE Randomized Clinical Trial, JAMA. (2019) 322, no. 4, 326–335. 31334795 10.1001/jama.2019.9346 PMC6652154

[8] Torbey M. T. , Pauls Q. , and Gentile N. , et al.Intensive Versus Standard Treatment of Hyperglycemia in Acute Ischemic Stroke Patient: A Randomized Clinical Trial Subgroups Analysis, Stroke. (2022) 53, no. 5, 1510–1515, 10.1161/STROKEAHA.120.033048. 35331007 PMC9022682

[9] Bellis A. , Mauro C. , Barbato E. , Ceriello A. , Cittadini A. , and Morisco C. , Stress-Induced Hyperglycaemia in Non-Diabetic Patients With Acute Coronary Syndrome: From Molecular Mechanisms to New Therapeutic Perspectives, International Journal of Molecular Sciences. (2021) 22, no. 2, 10.3390/ijms22020775, 775. 33466656 PMC7828822

[10] Kim S. , Park E. S. , Chen P. R. , and Kim E. , Dysregulated Hypothalamic-Pituitary-Adrenal Axis Is Associated With Increased Inflammation and Worse Outcomes After Ischemic Stroke in Diabetic Mice, Frontiers in Immunology. (2022) 13, 10.3389/fimmu.2022.864858, 864858. 35784349 PMC9243263

[11] Xie M. , Liu Z. , Dai F. , Cao Z. , and Wang X. , Predicting Stroke-Associated Pneumonia in Acute Ischemic Stroke: A Machine Learning Model Development and Validation Study with CBC-Derived Inflammatory Indices, International Journal of General Medicine. (2025) 18, 3117–3128, 10.2147/IJGM.S524450. 40529346 PMC12170845

[12] Felice F. , Sardelli G. , and Balestri F. , et al.Acute Hyperglycemia-Induced Inflammation in MIO-M1 Cells: The Role of Aldose Reductase, International Journal of Molecular Sciences. (2025) 26, no. 14, 10.3390/ijms26146741, 6741. 40724989 PMC12295778

[13] Dong L.-D. , Ma Y.-M. , and Xu J. , et al.Effect of Hyperglycemia on Microglial Polarization After Cerebral Ischemia-Reperfusion Injury in Rats, Life Sciences. (2021) 279, 10.1016/j.lfs.2021.119660, 119660. 34052292

[14] Wang H. , Deng J. L. , Chen L. , Ding K. , and Wang Y. , Acute Glucose Fluctuation Induces Inflammation and Neurons Apoptosis in Hippocampal Tissues of Diabetic Rats, Journal of Cellular Biochemistry. (2021) 122, no. 9, 1239–1247, 10.1002/jcb.29523. 31713299

[15] Feng X. , Yu F. , and Wei M. , et al.The Association Between Neutrophil Counts and Neutrophil-to-Lymphocyte Ratio and Stress Hyperglycemia in Patients With Acute Ischemic Stroke According to Stroke Etiology, Frontiers in Endocrinology. (2023) 14, 10.3389/fendo.2023.1117408, 1117408. 37008926 PMC10060840

[16] Ramezankhani A. , Tohidi M. , and Hadaegh F. , Association Between the Systemic Immune-Inflammation Index and Metabolic Syndrome and Its Components: Results From the Multi-Ethnic Study of Atherosclerosis (MESA), Cardiovascular Diabetology. (2025) 24, no. 1, 10.1186/s12933-025-02629-4, 78. 39955525 PMC11830208

[17] Zhang Y. , Xing Z. , Zhou K. , and Jiang S. , The Predictive Role of Systemic Inflammation Response Index (SIRI) in the Prognosis of Stroke Patients, Clinical Interventions in Aging. (2021) 16, 1997–2007, 10.2147/CIA.S339221. 34880606 PMC8645951

[18] Mo J. , Liu X. , and Zhang H. , et al.Inflammatory Burden Index and One-Year Clinical Outcomes in Large Artery Atherosclerosis Ischemic Stroke: A Multicenter Prospective Study, European Journal of Neurology. (2025) 32, no. 6, 10.1111/ene.70242, e70242. 40485600 PMC12146789

[19] Mendelson S. J. and Prabhakaran S. , Diagnosis and Management of Transient Ischemic Attack and Acute Ischemic Stroke: A Review, JAMA. (2021) 325, no. 11, 1088–1098, 10.1001/jama.2020.26867. 33724327

[20] Moghissi E. S. , Korytkowski M. T. , and DiNardo M. , et al.American Association of Clinical Endocrinologists and American Diabetes Association Consensus Statement on Inpatient Glycemic Control, Diabetes Care. (2009) 32, no. 6, 1119–1131, 10.2337/dc09-9029, 2-s2.0-66549097705. 19429873 PMC2681039

[21] Chen S. , Wan Y. , and Guo H. , et al.Diabetic and Stress-Induced Hyperglycemia in Spontaneous Intracerebral Hemorrhage: A Multicenter Prospective Cohort (CHEERY) Study, CNS Neuroscience & Therapeutics. (2023) 29, no. 4, 979–987, 10.1111/cns.14033. 36448225 PMC10018104

[22] Mifsud S. , Schembri E. L. , and Gruppetta M. , Stress-Induced Hyperglycaemia, British Journal of Hospital Medicine. (2018) 79, no. 11, 634–639, 10.12968/hmed.2018.79.11.634, 2-s2.0-85056329403. 30418830

[23] Ding L. , Zhang H. , and Dai C. , et al.The Prognostic Value of the Stress Hyperglycemia Ratio for All-Cause and Cardiovascular Mortality in Patients With Diabetes or Prediabetes: Insights From NHANES. 2005–2018, Cardiovascular Diabetology. (2024) 23, no. 1, 10.1186/s12933-024-02172-8, 84. 38419029 PMC10902955

[24] Ma H. , Chen R. , and Han N. , et al.Association Between Blood-Brain Barrier Disruption and Stroke-Associated Pneumonia in Acute Ischemic Stroke Patients After Endovascular Therapy: A Retrospective Cohort Study, Clinical Interventions in Aging. (2024) 19, 1611–1628, 10.2147/CIA.S475887. 39372167 PMC11453164

[25] Yuan X. , Huang S. , Ni J. , and Dong W. , Association Between Blood Triglycerides and Stroke-Associated Pneumonia: A Prospective Cohort Study, BMC Neurology. (2025) 25, no. 1, 10.1186/s12883-025-04060-4, 83. 40038569 PMC11877716

[26] Xu B. , Wu Q. , and La R. , et al.Is Systemic Inflammation a Missing Link Between Cardiometabolic Index With Mortality? Evidence From a Large Population-Based Study, Cardiovascular Diabetology. (2024) 23, no. 1, 10.1186/s12933-024-02251-w, 212. 38902748 PMC11191290

[27] Yao M. , Hao Y. , and Wang T. , et al.A Review of Stress-Induced Hyperglycaemia in the Context of Acute Ischaemic Stroke: Definition, Underlying Mechanisms, and the Status of Insulin Therapy, Frontiers in Neurology. (2023) 14, 10.3389/fneur.2023.1149671, 1149671. 37025208 PMC10070880

[28] Song X. , Ju Y. , and Gu H. , et al.Impact of Stress-Induced Hyperglycemia on In-Hospital Medical Complications in Patients With Acute Stroke: From a Large-Scale Nationwide Longitudinal Registry, Annals of Clinical and Translational Neurology. (2025) 16, 10.1002/acn3.70228. PMC12968442 41103093

[29] Elhefnawy M. , Nazifah Sidek N. , and Maisharah Sheikh Ghadzi S. , et al.Prevalence of Stroke-Associated Pneumonia and Its Predictors Among Hyperglycaemia Patients During Acute Ischemic Stroke, Cureus. (2024) 16, no. 1, 10.7759/cureus.52574, e52574. 38371076 PMC10874618

[30] Zonneveld T. P. , Nederkoorn P. J. , and Westendorp W. F. , et al.Hyperglycemia Predicts Poststroke Infections in Acute Ischemic Stroke, Neurology. (2017) 88, no. 15, 1415–1421, 10.1212/WNL.0000000000003811, 2-s2.0-85018625165. 28283600

[31] Jiang Z. , Wang K. , and Duan H. , et al.Association Between Stress Hyperglycemia Ratio and Prognosis in Acute Ischemic Stroke: A Systematic Review and Meta-Analysis, BMC Neurology. (2024) 24, no. 1, 10.1186/s12883-023-03519-6, 13. 38166660 PMC10759321

[32] Wang L. , Chen Y. , and Liu L. , et al.Influence of Glycated Hemoglobin on Thromboinflammation in Acute Ischemic Stroke: A Retrospective, Propensity Score Matching Study, Frontiers in Endocrinology. (2025) 16, 10.3389/fendo.2025.1542549, 1542549. 40791995 PMC12336038

[33] Cui Z. , Kuang S. , and Yang X. , et al.Predictive Value of the Systemic Immune Inflammation (SII) Index for Stroke-Associated Pneumonia, Brain and Behavior. (2023) 13, no. 12, 10.1002/brb3.3302, e3302. 37938870 PMC10726822

[34] Zheng F. , Gao W. , and Xiao Y. , et al.Systemic Inflammatory Response Index as a Predictor of Stroke-Associated Pneumonia in Patients With Acute Ischemic Stroke Treated by Thrombectomy: A Retrospective Study, BMC Neurology. (2024) 24, no. 1, 10.1186/s12883-024-03783-0, 287. 39148021 PMC11325834

[35] Duan T. , Yang M. , Zhang Y. , Zhu C. , and Rao Z. , Elevated Systemic Immune-Inflammation Index Is Associated With Stroke-Associated Pneumonia in Acute Ischemic Stroke: A Retrospective Cohort Study, Frontiers in Neurology. (2025) 16, 10.3389/fneur.2025.1651656, 1651656. 41059512 PMC12497611

[36] Hoffmann S. , Harms H. , and Ulm L. , et al.Stroke-Induced Immunodepression and Dysphagia Independently Predict Stroke-Associated Pneumonia—The PREDICT Study, Journal of Cerebral Blood Flow & Metabolism. (2017) 37, no. 12, 3671–3682, 10.1177/0271678X16671964, 2-s2.0-85036648449. 27733675 PMC5718319

[37] Berbudi A. , Rahmadika N. , Tjahjadi A. I. , and Ruslami R. , Type 2 Diabetes and Its Impact on the Immune System, Current Diabetes Reviews. (2020) 16, no. 5, 442–449, 10.2174/18756417MTAxgODQqy. 31657690 PMC7475801

[38] Hotamisligil G. S. , Inflammation and Metabolic Disorders, Nature. (2006) 444, no. 7121, 860–867, 10.1038/nature05485, 2-s2.0-33845866857. 17167474

[39] Elkind M. S. V. , Veltkamp R. , and Montaner J. , et al.Natalizumab in Acute Ischemic Stroke (ACTION II): A Randomized, Placebo-Controlled Trial, Neurology. (2020) 95, no. 8, e1091–e104, 10.1212/WNL.0000000000010038. 32591475 PMC7668547

[40] Hernandez-Jimenez M. , Abad-Santos F. , and Cotgreave I. , et al.Safety and Efficacy of ApTOLL in Patients With Ischemic Stroke Undergoing Endovascular Treatment: A Phase 1/2 Randomized Clinical Trial, JAMA Neurology. (2023) 80, no. 8, 779–788, 10.1001/jamaneurol.2023.1660. 37338893 PMC10282959

[41] Maes L. , Walsh C. , and Weimar C. , et al.Effect of Colchicine for Secondary Prevention According to Stroke Subtype: A Secondary Analysis of the CONVINCE Randomized Trial, International Journal of Stroke. (2025) 10.1177/17474930251406818, 17474930251406818. 41328787

[42] Akl E. , Sahami N. , and Labos C. , et al.Meta-Analysis of Randomized Trials: Efficacy and Safety of Colchicine for Secondary Prevention of Cardiovascular Disease, Journal of Interventional Cardiology. (2024) 2024, 10.1155/2024/8646351, 8646351. 38505729 PMC10950412

