# Supplementary material for: Systemic Inflammation Mediates the Association Between Admission Hyperglycemia and Pulmonary Infection or Prognosis in Acute Ischemic Stroke
Source: Mediators Inflamm. 2026 Mar 18;2026:9595535. doi: 10.1155/mi/9595535 (PMC13140444; doi:10.1155/mi/9595535)
Supplement: Supplementary file 1 — Supporting Information 1 The supporting information provide further data analysis on the included patients and subgroup logistic analysis between systemic inflammation and clinical outcome to support the main analysis. Table S1: Comparison of baseline characteristics between the included and excluded patients. Table S2: Logistic analysis of the association between systemic inflammation level with the risk of SAP in various glycemia conditions. Table S3: Logistic analysis of the association between systemic inflammation level with the risk of 12‐month poor prognosis in various glycemia conditions. [file MI-2026-9595535-s002.docx]

**Table S1 Comparison of baseline characteristics between the included and excluded patients**

| Characteristics | Included patients (n=2233) | Excluded patients (n=2519) | *P* |
| --- | --- | --- | --- |
| Age (years) | 65 (56, 73) | 66 (57, 76) | *0.091* |
| Gender (male, %) | 1523 (68.2%) | 1664 (66.1%) | *0.116* |
| BMI (kg/m^2^) | 24.22 (22.23, 26.12) | 24.00 (21.80, 26.12) | *0.001* |
| Smoking | 910 (40.8%) | 1005 (39.9%) | 0.548 |
| Alcohol | 553 (24.8%) | 685 (27.2%) | *0.057* |
| Medical history  Hypertension  Diabetes  Dyslipidaemia  Atrial fibrillation  Coronary heart disease  Stroke | 1394 (62.4%)  593 (26.6%)  451 (20.2%)  262 (11.8%)  380 (17.0%)  433 (19.4%) | 1597(63.4%)  681 (27.0%)  529 (21.0%)  193 (7.7%)  510 (20.2%)  465(18.5%) | *0.489*  *0.710*  *0.495*  *<0.001*  *0.004*  *0.413* |
| Admission mRS | 2 (1, 4) | 0 (0, 2) | <0.001 |
| Admission NIHSS | 4 (2, 9) | 0 (0, 3) | <0.001 |
| Heart rate | 75 (67, 84) | 76 (68, 85) | 0.085 |
| SBP (mmHg) | 150 (135, 164) | 146 (132, 162) | *<0.001* |
| DBP (mmHg) | 85 (76, 95) | 85 (76, 94) | *0.644* |
| Laboratory parameters  TC (mmol/L)  TG (mmol/L)  LDL (mmol/L)  HDL (mmol/L)  HbA1c (%)  Glucose (mmol/L)  Serum creatinine (U mol/L)  Blood urea nitrogen  Uric acid  Homocysteine (mmol/L)  WBC (×10^9^/L)  RBC (×10^12^/L)  PLT (×10^9^/L) | 4.18 (3.42, 4.87)  1.33 (0.93, 1.94)  2.35 (1.79, 2.89)  1.13 (0.95, 1.31)  5.9 (5.5, 6.9)  6.49 (5.46, 8.56)  64 (54, 75)  5.18 (4.16, 6.46)  323 (266, 386.25)  16 (12, 23)  7.08 (5.83, 8.71)  4.60 (4.15, 4.97)  193 (156, 234) | 4.10 (3.42, 4.82)  1.35 (0.95, 1.88)  2.48 (1.91, 3.09)  1.15 (0.98, 1.35)  6.0 (5.4, 6.8)  6.56 (5.52, 8.75)  67 (56, 79)  5.12 (4.12, 6.39)  319 (261, 384)  16 (12, 24)  7.12 (5.88, 8.95)  4.58(4.16, 4.95)  192 (157, 231) | *0.943*  *0.004*  *<0.001*  *0.989*  *0.393*  *0.804*  *<0.001*  *0.726*  *0.391*  *0.062*  *<0.001*  *0.529*  *0.623* |
| In-hospital days | 10 (8, 13) | 10 (7, 14) | 0.942 |

**Table S2 Logistic analysis of the association between systemic inflammation level with the risk of SAP in various glycemia conditions**

| Groups | Variables | Crude OR (95%CI) | *P* | Adjusted OR^*^ (95%CI) | *P* |
| --- | --- | --- | --- | --- | --- |
| NG (n=1341) | lnSII |  |  |  |  |
|  | T1 (≤ 6.004) (n=469) | reference |  | reference |  |
|  | T2 (6.005-6.653) (n=470) | 1.342 (0.866-2.080) | 0.188 | 1.160 (0.661-2.036) | 0.605 |
|  | T3 ( ≥ 6.654) (n=402) | 4.364 (2.946-6.466) | <0.001 | 3.158 (1.902-5.246) | <0.001 |
|  | SIRI |  |  |  |  |
|  | T1 (≤0.951) (n=486) | reference |  | reference |  |
|  | T2 (0.952-1.781) (n=447) | 1.124 (0.729-1.733) | 0.596 | 1.347 (0.780-2.328) | 0.285 |
|  | T3 ( ≥1.782) (n=408) | 3.754 (2.578-5.465) | <0.001 | 2.886 (1.779-4.682) | <0.001 |
| PHG  (n=588) | lnSII |  |  |  |  |
|  | T1 (≤ 6.004) (n=199) | reference |  | reference |  |
|  | T2 (6.005-6.653) (n=198) | 1.006 (0.550-1.839) | 0.985 | 0.841 (0.398-1.778) | 0.651 |
|  | T3 ( ≥ 6.654) (n=191) | 3.505 (2.077-5.914) | <0.001 | 2.143 (1.094-4.199) | 0.026 |
|  | SIRI |  |  |  |  |
|  | T1 (≤0.951) (n=189) | reference |  | reference |  |
|  | T2 (0.952-1.781) (n=205) | 1.151(0.631-2.101) | 0.646 | 1.306 (0.613-2.781) | 0.489 |
|  | T3 ( ≥1.782) (n=194) | 3.482 (2.033-5.963) | <0.001 | 2.899 (1.437-5.850) | 0.003 |
| SIH (n=304) | lnSII |  |  |  |  |
|  | T1 (≤ 6.004) (n=77) | reference |  | reference |  |
|  | T2 (6.005-6.653) (n=76) | 1.307 (0.654-2.614) | 0.449 | 1.293 (0.520-3.213) | 0.580 |
|  | T3 ( ≥ 6.654) (n=151) | 2.926 (1.615-5.302) | <0.001 | 2.227 (1.001-4.970) | 0.049 |
|  | SIRI |  |  |  |  |
|  | T1 (≤0.951) (n=70) | reference |  | reference |  |
|  | T2 (0.952-1.781) (n=92) | 0.919 (0.464-1.821) | 0.809 | 1.038 (0.425-2.535) | 0.934 |
|  | T3 ( ≥1.782) (n=142) | 2.844 (1.547-5.227) | 0.001 | 3.309 (1.341-6.888) | 0.008 |

^*^Adjusted for age, admission mRS and NIHSS, TOAST subtypes, clinical treatments and dysphagia.

**Table S3 Logistic analysis of the association between systemic inflammation level with the risk of 12-month poor prognosis in various glycemia conditions**

| Groups | Variables | Crude OR (95%CI) | *P* | Adjusted OR (95%CI)^*^ | *P* |
| --- | --- | --- | --- | --- | --- |
| NG  (n=1341) | lnSII |  |  |  |  |
|  | T1 (≤ 6.004) (n=469) | reference |  | reference |  |
|  | T2 (6.005-6.653) (n=470) | 1.183 (0.861-1.627) | 0.300 | 1.145 (0.787-1.667) | 0.479 |
|  | T3 ( ≥ 6.654) (n=402) | 2.159 (1.583-2.943) | <0.001 | 1.344 (0.917-1.971) | 0.130 |
|  | SIRI |  |  |  |  |
|  | T1 (≤0.951) (n=486) | reference |  | reference |  |
|  | T2 (0.952-1.781) (n=447) | 0.863 (0.622-1.198) | 0.535 | 0.818 (0.557-1.199) | 0.501 |
|  | T3 ( ≥1.782) (n=408) | 2.254 (1.670-3.041) | <0.001 | 1.441 (0.999-2.078) | 0.051 |
| PHG  (n=588) | lnSII |  |  |  |  |
|  | T1 (≤ 6.004) (n=199) | reference |  | reference |  |
|  | T2 (6.005-6.653) (n=198) | 1.323 (0.854-2.049) | 0.210 | 1.287 (0.783-2.117) | 0.320 |
|  | T3 ( ≥ 6.654) (n=191) | 2.092 (1.362-3.211) | 0.001 | 1.432 (0.852-2.407) | 0.175 |
|  | SIRI |  |  |  |  |
|  | T1 (≤0.951) (n=189) | reference |  | reference |  |
|  | T2 (0.952-1.781) (n=205) | 1.291 (0.834-1.998) | 0.252 | 1.477 (0.894-2.440) | 0.128 |
|  | T3 ( ≥1.782) (n=194) | 1.869 (1.213-2.880) | 0.005 | 1.345 (0.796-2.273) | 0.267 |
| SIH (n=304) | lnSII |  |  |  |  |
|  | T1 (≤ 6.004) (n=77) | reference |  | reference |  |
|  | T2 (6.005-6.653) (n=76) | 1.139 (0.597-2.174) | 0.692 | 1.014 (0.457-2.248) | 0.973 |
|  | T3 ( ≥ 6.654) (n=151) | 2.311 (1.318-4.053) | 0.003 | 2.047 (1.011-4.147) | 0.047 |
|  | SIRI |  |  |  |  |
|  | T1 (≤0.951) (n=70) | reference |  | reference |  |
|  | T2 (0.952-1.781) (n=92) | 1.245 (0.658-2.355) | 0.500 | 1.856 (0.807-4.271) | 0.146 |
|  | T3 ( ≥1.782) (n=142) | 2.677 (1.483-4.833) | 0.001 | 2.645 (1.196-5.848) | 0.016 |

*Adjusted for age, previous stroke history and mRS, admission mRS and NIHSS, awake stroke, dysphagia
