# Supplementary material for: Systemic Inflammation Mediates the Association Between Admission Hyperglycemia and Pulmonary Infection or Prognosis in Acute Ischemic Stroke
Source: Mediators Inflamm. 2026 Mar 18;2026:9595535. doi: 10.1155/mi/9595535 (PMC13140444; doi:10.1155/mi/9595535)
Supplement: Supplementary file 2 — Supporting Information 2 Figure S1: mRS distribution at 12‐month follow‐up in acute ischemic stroke patients stratified by admission glycemic status. [file MI-2026-9595535-s001.pdf]

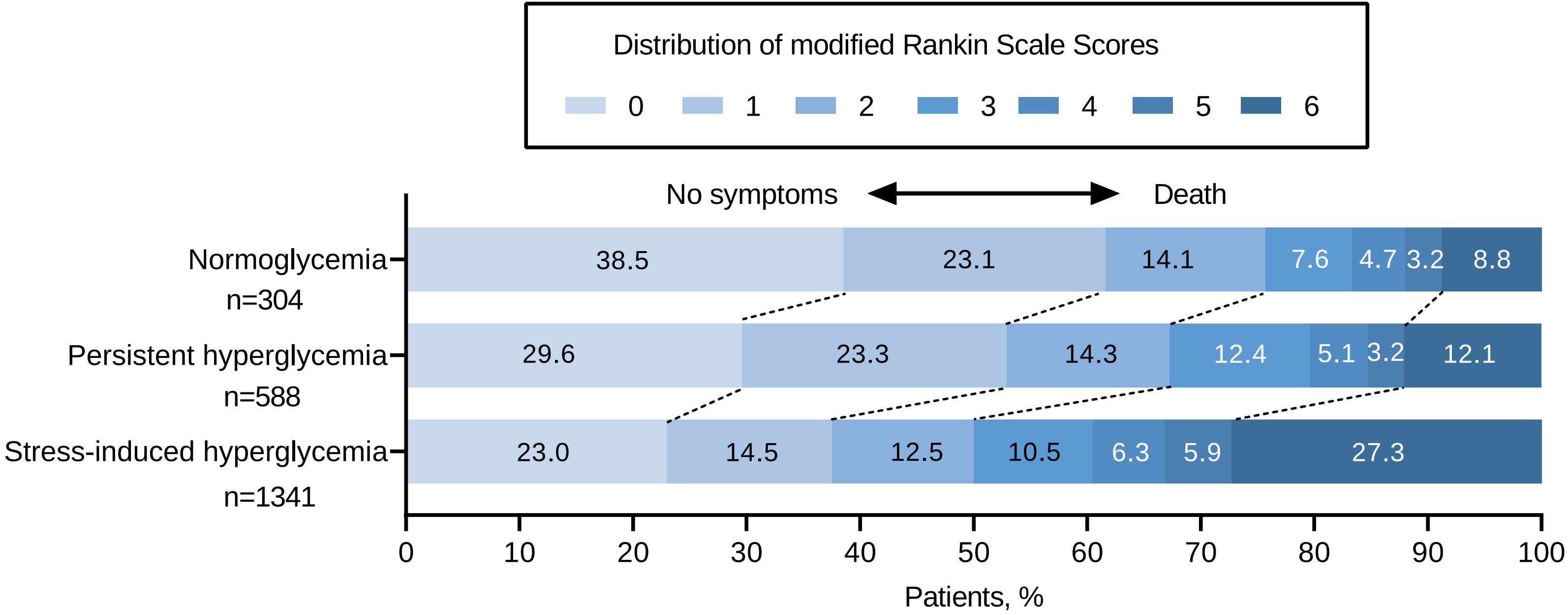

Distribution of modified Rankin Scale Scores
0
1
2
3
4
5
6
No symptoms
Death
Normoglycemia
n=304
38.5
23.1
14.1
7.6
4.7
3.2
8.8
Persistent hyperglycemia
n=588
29.6
23.3
14.3
12.4
5.1
3.2
12.1
Stress-induced hyperglycemia
n=1341
23.0
14.5
12.5
10.5
6.3
5.9
27.3
0
10
20
30
40
50
60
70
80
90
100
Patients, %
