# Supplementary figures and images for: Systemic Inflammation Mediates the Association Between Admission Hyperglycemia and Pulmonary Infection or Prognosis in Acute Ischemic Stroke
Source: Mediators Inflamm. 2026 Mar 18;2026:9595535. doi: 10.1155/mi/9595535 (PMC13140444; doi:10.1155/mi/9595535)

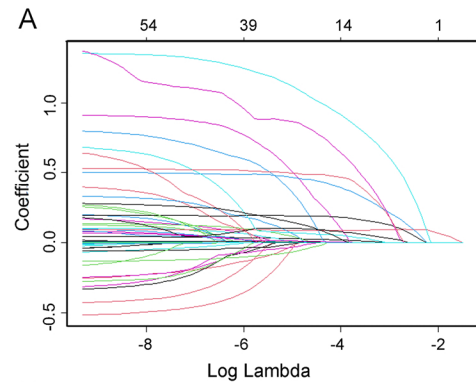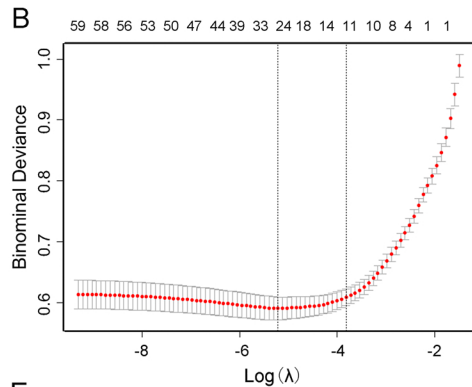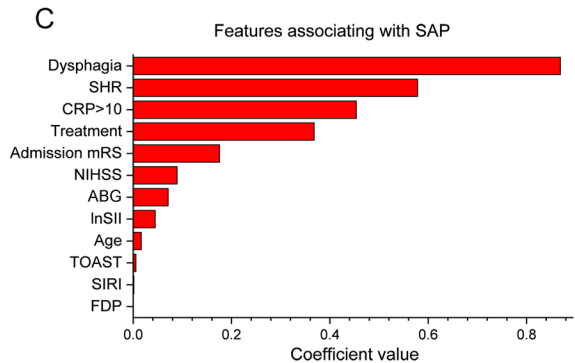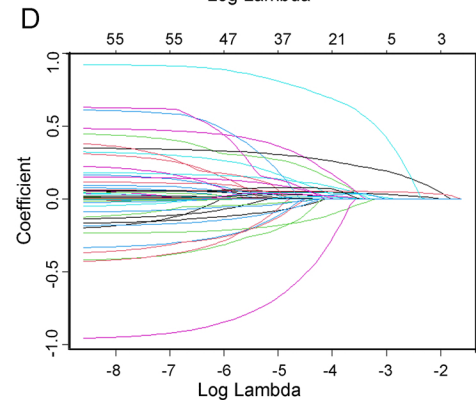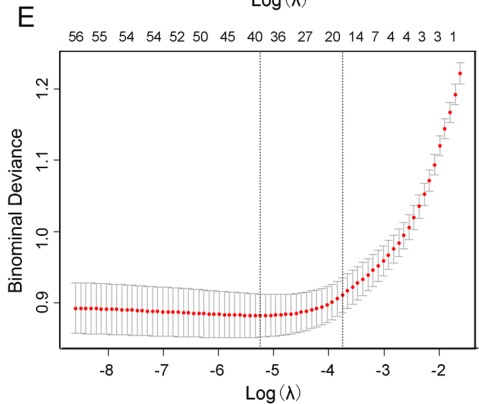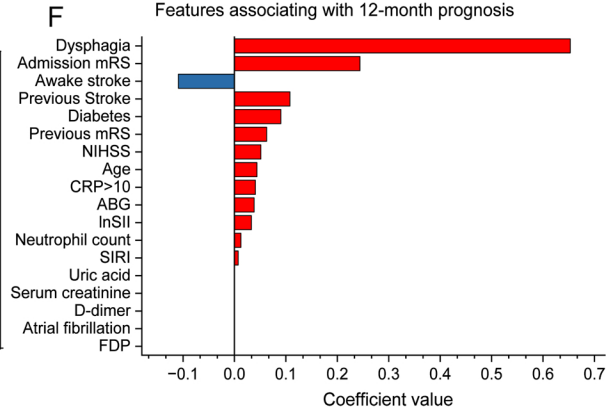

Supplement: Supplementary file 3 — Supporting Information 3 Figure S2: Clinical covariates selection for SAP or poor prognosis based on LASSO regression model. (A) The coefficient paths of all baseline clinical variables for SAP across a sequence of increasing log (λ) values, generated via 10‐fold cross‐validation. Each colored line represents the trajectory of a standardized coefficient for one variable. (B) Cross‐validation for optimal λ selection in LASSO regression for SAP. The mean cross‐validated deviance is plotted against log (λ). The left and right dashed vertical line marks the λ value at λ1min and λ1s, and the number of nonzero coefficients at each λ is shown along the top axis. (C) Predictor importance in the final sparse LASSO model. Horizontal bar plot displaying the standardized coefficients of the 8 features retained by the LASSO regression at the optimal λ1se. Variables are ranked by the absolute coefficients with red bar indicating a positive association with SAP while blue bar indicating an inverse association. (D–F) The procedure of feature selection associating with 12‐month poor prognosis using the same approach as above. [file MI-2026-9595535-s003.pdf]
